# Partial Reversal of Anosmia Following Platinum-Based Chemotherapy in a Patient With Colorectal Adenocarcinoma

**DOI:** 10.7759/cureus.44727

**Published:** 2023-09-05

**Authors:** Aditya Mahadevan, Armon Azizi, Naveen Bhandarkar, Farshid Dayyani

**Affiliations:** 1 Department of Hematology and Oncology, University of California Irvine School of Medicine, Orange, USA; 2 Department of Otolaryngology, University of California Irvine Medical Center, Orange, USA; 3 Department of Hematology and Oncology, University of California Irvine Medical Center, Orange, USA

**Keywords:** anosmia, taste and smell changes, caspase, olfactory disturbance, olfactory dysfunction, nasal septal polyp, olfaction disorders, oxaliplatin

## Abstract

Platinum-based chemotherapy is known to cause taste and smell changes (TSCs) via a host of mechanisms, including altered receptor activity, saliva/mucus production, and induction of receptor destruction via mitotic inhibition. In the literature to date, these changes have primarily resulted in worsening of taste and smell.

In this case report, we document the first instance of an individual regaining their sense of olfactory detection following treatment with oxaliplatin for colorectal adenocarcinoma. We theorize that the improvement in his sense of smell may have resulted from oxaliplatin-induced destruction of his nasal polyps through the caspase-9/procaspase-9 apoptotic pathway, a pathway shared with other mechanisms of nasal polyp destruction. These findings were supported by nasal endoscopy and sphenoid sinusoscopy, which demonstrated no clinical persistence of nasal polyps, in contrast to nasal endoscopy prior to chemotherapy which demonstrated persistent nasal polyposis. Objective smell testing post-treatment revealed a diminished ability to discriminate odors.

Chemotherapy-induced TSCs play a key role in poor weight gain, food aversion, emotional distress, and an overall decrease in quality of life, and patients should be informed of these potential consequences prior to starting treatment. However, in patients with anosmia secondary to nasal polyposis, treatment with platinum-based chemotherapy may provide an additional therapeutic benefit. Further studies may help elucidate the potential therapeutic benefits of these agents in managing steroid-resistant polyposis for patients suffering from olfactory dysfunction.

## Introduction

Oxaliplatin is a platinum-based chemotherapy used to treat colorectal cancer. Its anti-cancer mechanism is primarily derived through the cross-linking of DNA, leading to the arrest of DNA synthesis, inhibition of cell division, and death of rapidly dividing cell populations. Platinum-based chemotherapy drugs are associated with several side effects, including hepatotoxicity, anemia, and neuropathy, among others [1]. The changes in peripheral nervous system function are thought to be mediated by the release of reactive oxygen species, altering the activity of ion channels and mediating the release of proinflammatory cytokines [2]. More specifically, platinum-based chemotherapy has been linked to alterations in smell threshold, salt threshold, and food preference [3].

Taste and smell changes (TSCs), particularly deficits in taste and smell, have been associated with platinum-based chemotherapy across a range of cancer types [4,5]. There are several proposed mechanisms of chemotherapy-induced deficits in taste and smell, including altered receptor activity, saliva/mucus production, and induction of receptor destruction via mitotic inhibition [6-8]. Furthermore, other studies have hypothesized that these agents modify or damage the myelination state of cranial nerves, resulting in TSCs [9]. While many mechanisms have been proposed, the current literature consists of inconsistent and subjective evidence regarding TSCs, suggesting that further studies and data are needed in this area of research [9].

In this study, we present the case of a 74-year-old male patient with chronic anosmia who partially regained his sense of smell following treatment with four cycles of oxaliplatin for colorectal adenocarcinoma.

## Case presentation

**Figure 1:**
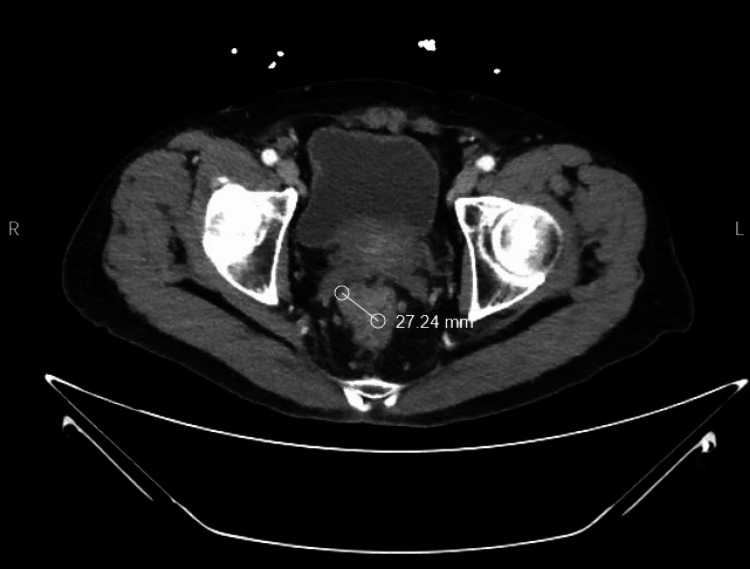
CT abdomen and pelvis demonstrating mid-rectal mass

A 74-year-old male with a past medical history of diabetes mellitus, hypothyroidism, glaucoma, asthma, nasal polyps, and chronic anosmia of more than 20 years was diagnosed with cT3N1M0 colorectal adenocarcinoma. The adenocarcinoma was diagnosed from a colonoscopy with a biopsy performed due to episodes of hematochezia. Subsequent CT confirmed a mass in the right lateral quadrant of the mid-rectum with evidence of perirectal invasion and enlargement of pelvic lymph nodes (Figure 1). Prior to further evaluation, the patient gave written consent.

**Figure 2:**
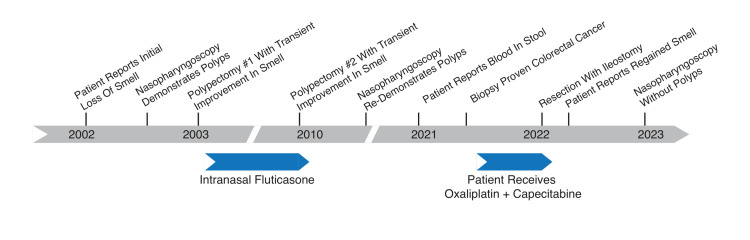
Timeline of patient's symptoms and interventions

Regarding the patient’s anosmia, he lost his sense of smell approximately 20 years prior to the diagnosis of colorectal cancer (Figure 2). During that time, the patient was working at a wastewater treatment plant where he was exposed to chlorine, hydrogen sulfide, and other chemical processes. He experienced severe congestion and underwent two nasal polyp surgeries within the span of 10 years. The patient was using intranasal fluticasone regularly between the surgeries. Despite these interventions, he noticed that he could “no longer smell his coffee” and that, eventually, his sense of smell was completely eliminated. The patient described testing his sense of smell during this time by placing an ammonia-based cleaning solution under his nose; he could not smell the solution at all, even though his eyes and nose were watering. Nasopharyngoscopy performed following his second polypectomy re-demonstrated nasal polyposis. His loss of smell persisted for over two decades with the exception of mild transient improvement in olfaction following both nasal polypectomies.

Following his cancer diagnosis, the patient received one month of chemoradiation with systemic capecitabine and localized radiation to the tumor. He subsequently began systemic therapy with a combination of capecitabine and oxaliplatin, which was administered for a total of four cycles. The patient reported that he experienced intermittent constipation and diarrhea as well as mild peripheral neuropathy in both hands during his fourth cycle of oxaliplatin. Subsequent imaging and sigmoidoscopy revealed residual malignancy, and the patient underwent a laparoscopic/robotic low anterior resection diverting ileostomy with negative surgical margins.

The patient reported changes in his sense of smell approximately six months following the completion of the fourth and final cycle of a combination of oxaliplatin and capecitabine. He reported that one day, he started to detect the smell of coffee again. Within one week of these changes in smell, the patient began to experience changes in taste, accompanied by nausea with food. Following his initial return of olfaction, the patient’s sense of smell continued to improve. However, he continued to endorse subjective alterations/changes in smell and taste relative to his baseline prior to and during his loss of olfaction. During this time, he denied experiencing nasal congestion, which he had previously experienced when he had been diagnosed with nasal polyps.

Following the patient’s return to olfaction, an otolaryngology evaluation was performed to further characterize his sense of smell. Nasal endoscopy and sphenoid sinusoscopy were performed, which demonstrated no clinical persistence of nasal polyps. Olfactory testing demonstrated diminished ability in smell discrimination (UPSIT score of 14/40).

## Discussion

Chemotherapy has been implicated in disruptions in smell and taste due to several proposed mechanisms, including altered receptor activity, saliva/mucus production, and induction of receptor destruction via mitotic inhibition [6-8]. However, anecdotal evidence has emerged regarding the potential benefits of various forms of chemotherapy in regaining the sense of smell, primarily in patients with nasal polyps, which have been implicated in the loss of smell [10,11].

To our knowledge, this case report presents the first documented case of a patient on primarily platinum-based chemotherapy (oxaliplatin) regaining their sense of smell. Patients on platinum-based chemotherapy regimens typically experience worsening of objective measures of smell [12]. However, our patient suffered from steroid-refractory nasal polyps with accompanying anosmia, much like patients in the aforementioned study [10].

We hypothesize that the patient's regained sense of smell was likely due, in part, to the destruction of his nasal polyps via oxaliplatin therapy. Nasal polyposis has been correlated with a less sensitive sense of smell (higher olfactory threshold), with extreme cases resulting in anosmia [13]. In addition to having nasal polyposis, our patient met several criteria that correlated with a poor sense of smell, including a history of asthma and old age [14]. In addition, patients with nasal polyposis who underwent endoscopic sinus surgery showed significant postoperative olfactory improvement compared to patients without nasal polyps, further implicating nasal polyps in loss of smell [14]. While the patient reported a subjective improvement in olfactory detection, objective olfactory testing revealed diminished olfactory discrimination, which we hypothesize is related to platinum-based olfactory disruption. Similarly, our patient’s transient improvement of smell following both nasal polypectomies supports our assertion that his anosmia was due to nasal polyps.

Prior literature in which patients experienced chemotherapy suggested several potential mechanisms of olfactory improvement through reduction or elimination of nasal polyps, including modification of cytokine and adenosine release [10]. Given that methotrexate has been shown to inhibit adenosine deaminase in vivo and nasal polyps express a markedly elevated adenosine deaminase level compared to normal nasal turbinate tissue, it follows that chemotherapy targeting adenosine deaminase may mediate improvement in smell through destruction/inhibition of proliferation of nasal polyps [15,16].

While authors in the aforementioned study hypothesized that benefits were due to systemic methotrexate therapy, two of the three patients who experienced olfactory improvement had an alkylating agent (cyclophosphamide) in their chemotherapy regimen, which has a similar mechanism to oxaliplatin [10]. Both oxaliplatin and cyclophosphamide induce cell death via the caspase-9/procaspase-9 pathway, much like dexamethasone, which has been proven to induce apoptosis of nasal polyps via the same pathway [17-19]. The shared mechanisms of corticosteroids and oxaliplatin in the context of nasal polyposis, combined with nasal endoscopy findings demonstrating resolution of nasal polyps compared to the nasal endoscopy performed prior to chemotherapy, suggest that the patient's olfactory improvement may have been due to oxaliplatin-mediated polyp destruction.

While a reduction in the burden of nasal polyposis is our primary hypothesis for the patient’s improvement in olfaction, other previously proposed mechanisms of chemotherapy-induced TSCs cannot be discounted. These mechanisms include regeneration of olfactory nervous tissue following damage from chemotherapy, modification of the myelination state of olfactory nervous tissue, altered receptor activity, and immune-mediated mechanisms [6-9]. Further studies are needed to evaluate the contribution of these mechanisms to olfactory improvement.

This case report has some limitations, notably, that we were unable to obtain the explicit nasal endoscopy report demonstrating the persistence of nasal polyposis following nasal polypectomy and prior to the initiation of chemotherapy. While the patient did partially regain his sense of smell, his UPSIT score was not reflective of full olfactory recovery. Finally, a single case may not be representative of the broader population of patients undergoing platinum-based chemotherapy. More comprehensive studies with larger sample sizes and comparison groups are necessary to draw further conclusions.

## Conclusions

This case report demonstrates the restoration of olfaction following platinum-based chemotherapy through a possible mechanism of nasal polyp destruction. Further studies are needed to understand the mechanism by which platinum-based chemotherapy agents may contribute to the restoration of olfaction in patients with nasal polyps and anosmia. Further studies may help elucidate the potential therapeutic benefits of these agents in managing steroid-resistant polyposis for patients suffering from olfactory dysfunction.
